# Diabetic Ketoacidosis Associated With the Menstrual Cycle: A Case of Catamenial Diabetic Ketoacidosis

**DOI:** 10.7759/cureus.99191

**Published:** 2025-12-14

**Authors:** Reza Alavi, Casper Chen, Hector Armando Sanchez Garcia, Obiora Udeozo

**Affiliations:** 1 Internal Medicine, HCA Florida Bayonet Point Hospital, Hudson, USA; 2 Medicine, HCA Florida Bayonet Point Hospital, Hudson, USA; 3 Critical Care Medicine, HCA Florida Bayonet Point Hospital, Hudson, USA

**Keywords:** catamenial dka, diabetic ketoacidosis, hormonal insulin resistance, menstrual cycle, type 1 diabetes

## Abstract

Catamenial diabetic ketoacidosis (DKA) is an uncommon but clinically significant phenomenon characterized by recurrent DKA episodes temporally related to the menstrual cycle. Hormonal fluctuations, particularly luteal-phase progesterone surges that contribute to transient insulin resistance, appear to play a central role in this condition. We present the case of a 34-year-old woman with type 1 diabetes mellitus (T1DM) who developed recurrent DKA episodes coinciding with her menstrual periods. Extensive evaluation ruled out infection, noncompliance, and insulin pump malfunction. A clear temporal association between symptom onset and menstruation was established through review of menstrual and glucose logs. She was treated with intravenous fluids, potassium replacement, and insulin infusion per DKA protocol. Within 24 hours, her anion gap closed, bicarbonate normalized, and she was transitioned to subcutaneous insulin with endocrinology follow-up.

Patterns of glucose dysregulation linked to menstrual hormonal shifts can create cyclical susceptibility to ketosis and metabolic decompensation. In this context, rising luteal-phase progesterone and accompanying hormonal changes may enhance counterregulatory activity and impair glucose utilization. Recognizing this pattern is essential, as targeted strategies such as hormonal modulation or anticipatory insulin dose adjustments may help prevent recurrence. A focused approach that considers individualized physiologic variation can improve diagnostic accuracy and long-term management in affected patients.

## Introduction

Diabetic ketoacidosis (DKA) is a life-threatening complication of diabetes resulting from insulin deficiency and elevated counterregulatory hormones, leading to hyperglycemia, ketosis, and acidosis. Although commonly triggered by infection, insulin omission, or new onset diabetes, a rare cyclical form linked to menstrual hormonal fluctuations known as catamenial DKA has been observed [1-4]. Variations in estrogen and progesterone during the luteal phase can transiently increase insulin resistance, provoking hyperglycemia and ketosis [1,2]. This mechanism mirrors other catamenial disorders such as epilepsy and asthma, where hormonal changes affect physiological control. Often underrecognized and misattributed to nonadherence, catamenial DKA warrants greater awareness [2]. We present a case highlighting its pathophysiology, diagnostic approach, and management considerations.

## Case presentation

A 34-year-old woman with a past medical history of insulin-dependent type 1 diabetes mellitus (T1DM), recurrent DKA temporally associated with her menstrual cycle, dysmenorrhea, iron deficiency anemia, hematemesis, hiatal hernia, and diabetic gastroparesis presented to the emergency department with lethargy and coffee ground emesis. On arrival, she was awake but profoundly lethargic. She has experienced multiple prior DKA episodes coinciding with the onset of menses. At this presentation, she was three days into her menstrual period. She reported adherence to her insulin regimen. Additional symptoms included nausea, abdominal pain, tachypnea, and a nonproductive cough.

On admission, laboratory evaluation demonstrated severe hyperglycemia with a serum glucose level of 716 mg/dL, elevated β-hydroxybutyrate of 125 mg/dL, an increased anion gap of 35 mmol/L, and a markedly reduced bicarbonate level (<10 mmol/L), as summarized in Table 1. Arterial blood gas analysis demonstrated a PCO_2_ of 21.1 mmHg, as shown in Table 2. The patient was started on aggressive intravenous fluid resuscitation with lactated Ringer’s solution, and potassium was repleted prior to initiating insulin therapy. Continuous intravenous insulin infusion was then started along with potassium infusion. Once her glucose levels dropped below 250 mg/dL, dextrose infusion was added along with continuous insulin. Electrolytes and glucose levels were monitored frequently. Within 24 hours of initiating therapy, the patient’s anion gap closed, bicarbonate levels normalized, and she was able to tolerate oral intake. The insulin drip was transitioned to her subcutaneous insulin regimen. She was discharged in stable condition with close endocrinology follow-up.

**Table 1:** Basic Metabolic Panel (BMP) Laboratory findings on admission demonstrated significant hyperglycemia, elevated anion gap metabolic acidosis, and ketosis. Abnormal values are shown in bold in the original report. BUN: blood urea nitrogen; eGFR (CKD-EPI 2021): estimated glomerular filtration rate (Chronic Kidney Disease-Epidemiology collaboration 2021)

Test	Result	Normal Range
Sodium	136	135-145 mmol/L
Potassium	3.9	3.5-5.1 mmol/L
Chloride	91	98-107 mmol/L
Carbon Dioxide (CO_2_)	<10	22-29 mmol/L
Anion Gap	35	8-16 mmol/L
BUN	21	7-20 mg/dL
Creatinine	2.3	0.6-1.3 mg/dL
eGFR (CKD-EPI 2021)	27.9	>60 mL/min/1.73 m^2^
Glucose	716	70-99 mg/dL (fasting)
Lactic Acid	5.9	0.5-2.2 mmol/L
Beta-Hydroxybutyrate	125	<3 mg/dL (≈0.3 mmol/L)

**Table 2:** Arterial Blood Gas (ABG) Arterial blood gas on admission demonstrated high anion gap metabolic acidosis with superimposed respiratory alkalosis, reflected by a low pCO_2_ and reduced bicarbonate level relative to expected physiologic compensation. Abnormal values are shown in bold in the original report.

Parameter	Result	Normal Range
pH	7.466	7.35-7.45
pCO_2_	21.1 mmHg	35-45 mmHg
pO_2_	97.3 mmHg	80-100 mmHg
Bicarbonate	14.9 mmol/L	22-26 mmol/L
O_2_ Saturation	98.20%	95-100%
Carboxyhemoglobin	1.40%	0-2% (nonsmokers)
Oxyhemoglobin	96.50%	94-100%

## Discussion

The menstrual cycle exerts significant effects on glucose homeostasis, particularly in women with T1DM. During the luteal phase, elevated progesterone levels induce insulin resistance by reducing peripheral glucose uptake and enhancing hepatic gluconeogenesis, while estrogen fluctuations further contribute to glycemic instability [5-7]. This hormonal interplay often leads to transient premenstrual hyperglycemia that, if not addressed, can progress to ketosis or frank DKA [5,6]. Studies using continuous glucose monitoring have confirmed that women experience higher glucose variability and elevated fasting glucose during the luteal phase compared to the follicular phase [7,8].

Catamenial DKA represents an underrecognized subset of recurrent DKA precipitated by cyclical hormonal fluctuations rather than traditional triggers such as infection, insulin omission, or stress [1-4]. Reported cases have consistently demonstrated recurrence of DKA episodes correlating with the onset of menstruation [1,5]. Gomez et al. [5] and Gamarra and Trimboli [6] both highlighted that progesterone-driven insulin resistance and estrogen withdrawal collectively impair glycemic control, especially in the premenstrual period. Milionis et al. [8] further emphasized that even in well-controlled T1DM, luteal-phase insulin resistance may elevate β-hydroxybutyrate levels, increasing the risk for ketosis despite adherence to insulin therapy.

Management of recurrent catamenial DKA requires an anticipatory, multidisciplinary approach. Common precipitating factors such as infection, insulin pump malfunction, or psychosocial stress should first be excluded [3,4]. Once hormonal cyclicity is identified, individualized strategies may include basal insulin titration during the luteal phase, close glucose surveillance through continuous glucose monitoring, and menstrual tracking to preempt glycemic excursions [6-8]. Hormonal modulation with combined oral contraceptives has been proposed to blunt progesterone-induced insulin resistance and stabilize glycemic fluctuations [1,5,6]. Coordination between endocrinology and gynecology is crucial to achieving optimal long-term metabolic control and preventing recurrence [5-8].

## Conclusions

Catamenial DKA represents an intersection of endocrinology and reproductive physiology, where cyclic hormonal changes provoke transient insulin resistance severe enough to precipitate DKA. Awareness of this condition remains limited, leading to diagnostic delays and recurrent hospitalizations. Clinicians should maintain a high index of suspicion for DKA during the menstrual phase in women with T1DM and proactively adjust insulin regimens to mitigate this increased risk. Recognition of menstrual-linked glycemic variation, proactive insulin titration, and potential hormonal modulation are cornerstone interventions. Given the rarity of reported cases, further multicenter research is needed to define prevalence, hormonal thresholds, and standardized management protocols. Early identification and individualized treatment can prevent recurrence and significantly enhance metabolic stability in affected women.
